# Predictors of Acute Kidney Disease Severity in Hospitalized Patients with Acute Kidney Injury

**DOI:** 10.3390/biomedicines10051081

**Published:** 2022-05-06

**Authors:** Pai-Chin Hsu, Chih-Han Liu, Wen-Chin Lee, Chien-Hsing Wu, Chien-Te Lee, Chien-Hao Su, Yu-Chin Lily Wang, Kai-Fan Tsai, Terry Ting-Yu Chiou

**Affiliations:** 1 Division of Nephrology, Department of Internal Medicine, Kaohsiung Chang Gung Memorial Hospital and Chang Gung University College of Medicine, Kaohsiung 83301, Taiwan; nick9335@cgmh.org.tw (P.-C.H.); dlm0909@cgmh.org.tw (C.-H.L.); leewenchin@gmail.com (W.-C.L.); chienhsingwu@gmail.com (C.-H.W.); ctlee33@cgmh.org.tw (C.-T.L.); 2 Department of Pharmacy, Kaohsiung Chang Gung Memorial Hospital and Chang Gung University College of Medicine, Kaohsiung 83301, Taiwan; yeorno@cgmh.org.tw (C.-H.S.); lilywang@cgmh.org.tw (Y.-C.L.W.); 3 Chung Shan Medical University School of Medicine, Taichung 40201, Taiwan

**Keywords:** acute kidney disease, acute kidney injury, predictors, severity, offending drugs, mortality, dialysis

## Abstract

Acute kidney disease (AKD) forms part of the continuum of acute kidney injury (AKI) and worsens clinical outcomes. Currently, the predictors of AKD severity have yet to be established. We conducted a retrospective investigation involving 310 hospitalized patients with AKI and stratified them based on the AKD stages defined by the Acute Dialysis Quality Initiative criteria. Demographic, clinical, hematologic, and biochemical profiles, as well as 30-day outcomes, were compared between subgroups. In the analysis, the use of offending drugs (odds ratio, OR (95% confidence interval, CI), AKD stage 3 vs. non-AKD, 3.132 (1.304–7.526), *p* = 0.011, AKD stage 2 vs. non-AKD, 2.314 (1.049–5.107), *p* = 0.038), high AKI severity (OR (95% CI), AKD stage 3 vs. non-AKD, 6.214 (2.658–14.526), *p* < 0.001), and early dialysis requirement (OR (95% CI), AKD stage 3 vs. non-AKD, 3.366 (1.008–11.242), *p* = 0.049) were identified as independent predictors of AKD severity. Moreover, a higher AKD severity was associated with higher 30-day mortality and lower dialysis-independent survival rates. In conclusion, our study demonstrated that offending drug use, AKI severity, and early dialysis requirement were independent predictors of AKD severity, and high AKD severity had negative impact on post-AKI outcomes.

## 1. Introduction

Acute kidney injury (AKI) is a common clinical disorder that occurs in approximately 10–20% of all inpatients and as high as half of the critically ill patients. As an impactive disease, AKI is associated with a longer hospital stay, higher health care cost, and poor outcomes such as chronic kidney disease (CKD), cardiovascular events, and mortality [1,2,3,4,5,6]. In 2012, the Kidney Disease Improving Global Outcomes (KDIGO) guidelines defined AKI as an abrupt decline in renal function within 7 days and CKD as an abnormality of renal structure or function for more than 90 days [7,8]. Since AKI and CKD have been increasingly considered related and continuous entities, the concept of a continuum was then developed. Consequently, a standardized definition for the gap period between AKI and CKD is imperatively required [9]. In 2017, the Acute Dialysis Quality Initiative (ADQI) 16 Workgroup defined acute kidney disease (AKD) as a condition in which acute renal damage with a severity of KDIGO stage 1 or greater persists for ≥7 days after initial AKI insult [10]. Emerging evidence supports that AKD worsens renal outcome and post-AKI mortality. In a retrospective analysis of patients with septic AKI, AKD affected over 50% of the study population and worsened renal outcome and mortality by approximately 3 times and 1.5 times, respectively [11]. In another investigation of 2556 AKI patients, AKD was also positively associated with adverse renal events as well as higher 1-year mortality [12]. These findings highlight the importance of AKD in clinical management during the post-AKI period, and further research is needed to elucidate the risk factors, clinical course, prognosis, and treatment strategy for AKD. Despite the increased concern, there has been a relative paucity of literature investigating the risk factors of AKD and the predictors of AKD severity. In the current studies, age, male sex, AKI severity, etiologies of AKI, diabetes, anemia, and pre-existing kidney disease were proposed as risk factors for progression from AKI to AKD, but the predictors of AKD severity have been less investigated [13,14]. In addition, the relationship between AKD severity and aggravating factors, such as offending drugs and contrast exposure, has yet to be specifically clarified. Considering the clinical impact of AKD, the recognition of possible predictors of AKD severity is essential for this vulnerable population.

In this retrospective study, we assessed the relationship between AKI severity, AKD severity, and clinical outcomes and analyzed the demographic, clinical, hematologic, and biochemical predictors of AKD severity in hospitalized patients with AKI.

## 2. Materials and Methods

### 2.1. Patients

AKI patients living in geographically different areas in southern Taiwan were recruited from the database of the AKI electronic alert (e-Alert) system of Kaohsiung Chang Gung Memorial Hospital between January 2019 and December 2020. The AKI e-Alert system, which was modified from published designs to facilitate the real-time detection of AKI [15,16], has been introduced into hospital practice since 2017. This system can automatically identify AKI events in hospitalized patients in adherence to the KDIGO criteria, i.e., an increase in serum creatinine (SCr) ≥ 26.52 µmol/L or ≥1.5 times the previous level within 7 days [8], with an alert sent to the mobile phone of the primary care physician as well as a record in the database of the hospital. The data of all patients with records in the system during the study period were reviewed. For those with multiple AKI records during the same hospitalization period, only their first AKI event was evaluated. The inclusion criteria were as follows: (1) adult patients (age ≥20 years) with AKI insults and admitted to general medical wards and (2) patients with available SCr data between the 7th and 9th days after the initial AKI insult. Patients undergoing maintenance renal replacement therapy (RRT) such as hemodialysis and peritoneal dialysis, those with history of kidney transplantation, those undergoing operations within 1 week before and after AKI events, and those with active pregnancy were excluded. The study protocol was approved by the Institutional Review Board and Ethics Committee of Chang Gung Medical Foundation, Taipei, Taiwan (IRB No. 201802329B0, 201902059B0), and adhered to the principles of the Declaration of Helsinki and Declaration of Istanbul. Informed consent was waived due to the retrospective design and minimal risk of the study.

### 2.2. Definition and Staging of AKI and AKD

The AKI severity was assessed based on the KDIGO criteria (i.e., stage 1, stage 2, and stage 3 indicating increase in SCr level between 1.5–2, 2–3, and >3 times the previous levels within 7 days, respectively), and patients requiring urgent RRT initiation were also classified as KDIGO stage 3, as per previously established criteria [8]. The AKD definition proposed by the ADQI 16 Workgroup in 2017 (i.e., acute renal injury with a severity of KDIGO stage 1 or greater lasting ≥7 days after AKI diagnosis) [10] was used for AKD diagnosis based on the SCr levels between the 7th and 9th days after AKI insults. For those with several SCr values available during the period, the mean SCr levels were used for the assessment. The staging of AKD was also defined according to the ADQI 16 consensus (i.e., stage 1, stage 2, and stage 3 indicating persistent increase in SCr level between 1.5–2, 2–3, and >3 times the baseline levels before AKI insults, respectively) and was based on the same SCr level for AKD diagnosis.

### 2.3. Demographic Profiles, Clinical Characteristics, and Outcomes Collection

The demographic profiles and clinical characteristics of the enrolled patients were collected from the electronic medical record system of the hospital. These data included age, sex, body mass index, hemodynamic status, main etiologies of AKI, contrast exposure, offending drugs for renal injury, RRT requirement, and comorbidities (such as diabetes, hypertension, dyslipidemia, CKD, heart failure, vascular disease, liver disease, pulmonary disease, and malignancy). The collected data were reviewed by three consultant nephrologists. The main etiologies of AKI were classified into five categories: sepsis, cardiorenal syndrome, hypovolemia (such as bleeding and dehydration), obstructive uropathy (such as functional voiding difficulties and structural abnormalities of the urinary tract), and intrarenal causes (such as acute tubular necrosis, acute tubulointerstitial nephritis, glomerulonephritis, and intrarenal vasculitis). Shock was defined as the use of inotropics or vasopressors or presence of at least two consecutive records of mean arterial pressure below 65 mmHg within 24 h before and after AKI diagnosis. Contrast exposure was recorded if patients received intravascular iodinated contrast within 1 week before AKI insult. Offending drugs prescribed within 1 week before AKI were also recorded, including drugs with potential tubular toxicity such as non-steroidal anti-inflammatory drugs (NSAIDs), cyclooxygenase-2 inhibitors (COX-2Is), and some antineoplastic agents (cisplatin, methotrexate, cancer immunotherapy, etc.); medications interfering with intraglomerular hemodynamics such as angiotensin-converting enzyme inhibitors (ACEIs) and angiotensin receptor blockers (ARBs); and nephrotoxic antimicrobial agents such as aminoglycosides and colistin. RRT requirements within the 1st (0–6 days, before AKD assessment) and 2nd weeks of AKI (7–14 days, after AKD assessment) were documented. CKD was defined according to the KDIGO 2012 Clinical Practice Guidelines for the Evaluation and Management of CKD [7] and based on two consecutive SCr-based estimated glomerular filtration rate (eGFR) data (one within 7 days before AKI insult and another within 3 months prior to enrollment). The Modification of Diet in Renal Disease equation, which is eGFR (mL/min/1.73 m^2^) = 175 × SCr^−1.154^ × age^−0.203^ × 0.742 (if female), was used to calculate eGFR [17]. Diabetes was defined as the regular use of at least one glucose-lowering agent or having at least two consecutive measurements of glycated hemoglobin of ≥6.5%. Hypertension was defined as the regular use of at least one antihypertensive agent or having more than two blood pressure records above 140/90 mmHg in the outpatient department. Dyslipidemia was defined as the regular use of a lipid-lowering agent or having at least two consecutive tests revealing abnormal lipid profiles (i.e., total cholesterol ≥ 5.18 mmol/L, low-density lipoprotein cholesterol ≥ 3.37 mmol/L, high-density lipoprotein cholesterol ≤ 1.04 mmol/L, or triglyceride ≥ 1.69 mmol/L). A history of malignancy was recorded if patients were diagnosed with malignant solid tumors or hematologic disorders and were yet to be disease-free for >3 years. Data of other comorbidities such as vascular diseases (including cardiovascular, cerebrovascular, carotid, and peripheral vascular diseases), liver diseases (including hepatitis B infection, hepatitis C infection, and cirrhosis), pulmonary diseases (including chronic obstructive pulmonary disease and interstitial lung disease), and heart failure were extracted from medical records. Additionally, the 30-day clinical outcomes of the study population were collected for evaluation, including RRT-independent survival, RRT-dependent survival, and mortality. The presence of de novo CKD (i.e., persisted eGFR < 60 mL/min/1.73 m^2^ beyond 3 months after AKI in those without pre-existing CKD), the mortality events between 2 and 3 months after AKI, and the requirement of long-term dialysis over 3 months were also recorded for patients surviving beyond 30 days after AKI.

### 2.4. Assessment of Hematologic and Biochemical Profiles

The hematologic and biochemical profiles of enrolled AKI patients, including SCr, eGFR, hemoglobin, platelet count, blood urea nitrogen, potassium, albumin, lactate, blood arterial gas and total bilirubin levels, were all recorded on the day of AKI insult. In addition, eGFR levels at baseline and on the day of AKD assessment were collected for further analysis.

### 2.5. Statistical Analysis

The severity of AKI (stratified by KDIGO staging) and severity of AKD (stratified by ADQI criteria) were compared using the chi-square test and are presented as numbers with percentages. To evaluate the relationship between AKD staging, clinical outcomes, and the predictors of AKD severity, the study population was divided into four subgroups according to the AKD stages. The data from the four subgroups (i.e., non-AKD, AKD stage 1, AKD stage 2, and AKD stage 3) were compared and analyzed. Categorical variables were analyzed using the chi-square test and are presented as numbers with percentages. Since most data had non-normal distribution revealed by the Kolmogorov–Smirnov method, continuous variables are presented as medians with interquartile ranges (IQRs), and the Kruskal–Wallis H-test was performed for univariate analysis. Considering the sample size of the cohort, AKI KDIGO stage 1 and 2 were combined for comparison with KDIGO stage 3 in the analyses. All variables with a *p*-value of <0.10 in univariate analyses were assessed by the multinomial logistic regression analysis with the enter method to determine the independent predictors of AKD severity, adjusting for age, sex, hypertension, diabetes, and other covariates. Statistical significance was set at a *p*-value of <0.05. Statistical Product and Service Solutions (version 22.0; IBM, Armonk, NY, USA) was used for all analyses.

## 3. Results

### 3.1. Characteristics of Enrolled Patients

We enrolled 310 adults in this study, including patients with AKI KDIGO stage 1 (*n* = 15, 4.84%), KDIGO stage 2 (*n* = 213, 68.71%), and KDIGO stage 3 (*n* = 82, 26.45%). The characteristics of the enrolled patients are summarized in Table 1. The median age of the cohort was 69 (IQR, 58–79) years, and women accounted for 40.32% of all patients. There were 41.61% of patients with non-dialysis-dependent CKD, and the median eGFR at baseline was 71.65 (IQR, 41.76–105.43) mL/min/1.73 m^2^. The most common comorbidity was hypertension (52.58%), followed by malignancy (45.81%), diabetes (43.55%), liver disease (30.00%), vascular disease (25.16%), heart failure (17.42%), dyslipidemia (15.16%), and pulmonary disease (11.61%). Moreover, sepsis was the most common main cause of AKI (60.65%), and 23.87%, 7.10%, 4.19%, and 4.19% of AKI cases were attributed to intrarenal causes, cardiorenal syndrome, hypovolemia, and obstructive uropathy, respectively. At the time of AKI diagnosis, the median eGFR decreased to 22.53 (IQR, 12.06–35.38) mL/min/1.73 m^2^, which was compatible with the settings of acute renal injury. In the overall cohort, 12.90% and 12.26% of patients required RRT within the 1st and 2nd weeks of AKI, respectively, and 30-day mortality after AKI was 22.90%. All RRT modalities in the cohort were hemodialysis, initiated according to the judgment of consultant nephrologists of the hospital and performed in adherence to the 2012 KDIGO Clinical Practice Guideline for AKI [8]. The major cause of 30-day mortality in the study population was sepsis (52.11%), followed by malignancy-associated complications (23.94%), decompensated liver cirrhosis (11.27%), cardiovascular diseases (8.45%), and other causes (4.23%). In patients surviving beyond 30 days after AKI (*n* = 239), de novo CKD was found in 25.19% of those without pre-existing CKD (33 out of the 131 cases). In addition, there were 18.82% and 7.11% of the survivors with mortality between 2 and 3 months after AKI and requiring long-term dialysis over 3 months after AKI, respectively.

### 3.2. Relationship between AKI Severity, AKD Stage, and 30-Day Outcome after AKI

**Figure 1:**
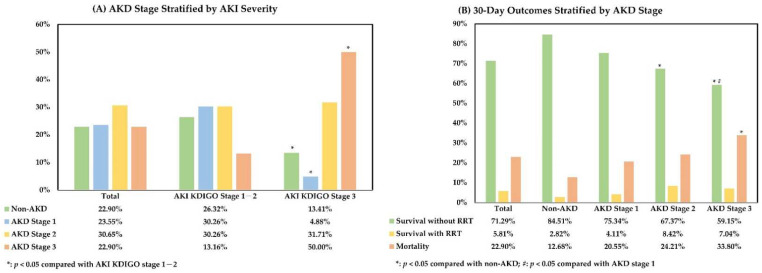
Relationship between AKI severity, AKD stage, and 30-day outcome after AKI. (**A**). AKD stage stratified by AKI severity; (**B**). 30-day outcomes stratified by AKD stage. All comparisons were performed using the chi-square test. AKD, acute kidney disease; AKI, acute kidney injury; KDIGO, Kidney Disease Improving Global Outcomes criteria; RRT, renal replacement therapy.

The AKD stages of the enrolled patients were defined based on the SCr levels between the 7th and 9th days after an AKI event. The relationship between AKI severity and AKD stage is shown in Figure 1A, which was assessed using the chi-square test. In the analysis, AKD stage 3 was more common in patients with AKI KDIGO stage 3 (KDIGO stage 1–2 vs. stage 3, 13.16% vs. 50.00%, *p* < 0.001), and patients with milder AKI were more frequently diagnosed with AKD stage 1 (KDIGO stage 1–2 vs. stage 3, 30.26% vs. 4.88%, *p* < 0.001) and non-AKD status (KDIGO stage 1–2 vs. stage 3, 26.32% vs. 13.41%, *p* = 0.017). For further evaluation, the cohort was divided into four subgroups according to the AKD stage, i.e., the non-AKD (*n* = 71, 22.90%), AKD stage 1 (*n* = 73, 23.55%), AKD stage 2 (*n* = 95, 30.65%), and AKD stage 3 (*n* = 71, 22.90%) subgroups. The median eGFR was 67.57 (IQR, 37.07–94.57), 29.91 (IQR, 13.93–47.32), 24.68 (IQR, 13.61–39.44), and 14.16 (IQR, 8.70–23.42) mL/min/1.73 m^2^ in the non-AKD, AKD stage 1, AKD stage 2, and AKD stage 3 subgroups, respectively. In the comparison using the chi-square test, the 30-day mortality after AKI was significantly higher in the AKD stage 3 subgroup (12.68%, 20.55%, 24.21%, and 33.80%, *p* = 0.026). In addition, the AKD stage 2 and AKD stage 3 subgroups had significantly fewer patients surviving without RRT support (84.51%, 75.34%, 67.37%, and 59.15%, *p* = 0.006) (Figure 1B). In patients surviving beyond 30 days after AKI, the presence of de novo CKD also increased with AKD severity (10.00%, 20.00%, 36.11%, and 36.67% of those without pre-existing CKD, *p* = 0.022). On the other hand, the mortality events between 2 and 3 months after AKI seemed more common in AKD stage 2 and stage 3 subgroups (9.68%, 15.52%, 26.39%, and 23.40%, *p* = 0.067), and there were 4.84%, 12.07%, 8.33%, and 2.13% of patients requiring long-term dialysis over 3 months after AKI in the non-AKD and AKD stage 1–3 subgroups, respectively (*p* = 0.206). In summary, our analysis indicated that AKI severity was positively correlated with AKD severity and that a high AKD severity was a risk factor for 30-day mortality with a negative impact on the renal outcomes after AKI.

### 3.3. Factors Associated with AKD Severity in Patients with AKI

To identify the predictors of AKD severity, demographic profiles, comorbidities, main AKI etiologies, AKI severity, aggravating factors for renal injury, hematologic data, and biochemical profiles were compared between AKD subgroups (Table 2 and Table 3). In univariate analyses, there were more patients with underlying heart failure (15.49%, 27.40%, 18.95%, and 7.04%, respectively, *p* = 0.013) and CKD (35.21%, 54.79%, 43.16%, and 32.39%, respectively, *p* = 0.029) in the AKD stage 1 subgroup, and hypovolemia was slightly more common as the main AKI etiology in the non-AKD subgroup (9.86%, 1.37%, 3.16%, and 2.82%, *p* = 0.052). Moreover, the use of offending drugs was more frequent in the AKD stage 3 subgroup (16.90%, 32.88%, 34.74%, and 36.62%, *p* = 0.038), and AKI severity was significantly higher in AKD stage 2 and stage 3 subgroups (AKI KDIGO stages 1–2, 84.51%, 94.52%, 72.63%, and 42.25%, *p* < 0.001; AKI KDIGO stage 3, 15.49%, 5.48%, 27.37%, and 57.75%, *p* < 0.001). Among the offending drugs, the use of antineoplastic agents or nephrotoxic antibiotics increased with the stages of AKD (2.82%, 5.48%, 7.37%, and 15.49%, *p* = 0.030). Additionally, the RRT requirement within the 1st week of AKI seemed more common in the AKD stage 3 subgroup (8.45%, 15.07%, 8.42%, and 21.13%, *p* = 0.057), and the baseline eGFR was lower in the AKD stage 1 subgroup (median (IQR), 81.31 (50.28–107.18), 56.90 (27.82–90.71), 70.18 (38.76–101.99), and 85.14 (57.32–127.95) mL/min/1.73 m^2^, *p* = 0.003). Other demographic data, comorbidities, main AKI etiologies, aggravating factors, hematologic data, and biochemical profiles were not significantly different between subgroups.

### 3.4. Independent Predictors of AKD Severity in Patients with AKI

**Figure 2:**
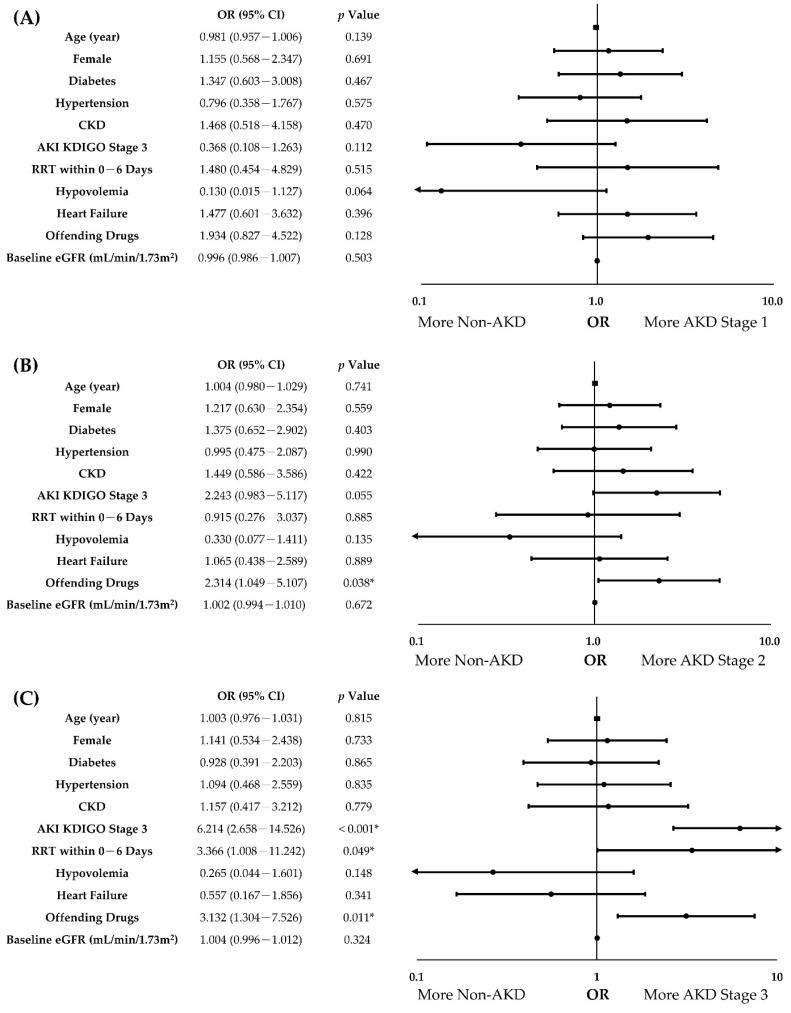
Forest plots of independent predictors of AKD severity in patients with AKI. (**A**). AKD Stage 1 vs. non-AKD; (**B**). AKD Stage 2 vs. non-AKD; (**C**). AKD Stage 3 vs. non-AKD. Age, sex, diabetes, hypertension, and covariates with a *p*-value of <0.10 in univariate analyses were adjusted for multinomial logistic regression analysis with the enter method. CI, confidence interval; CKD, chronic kidney disease; eGFR, estimated glomerular filtration rate; OR, odds ratio. *: *p* < 0.05.

To determine the independent predictors of AKD severity, all covariates with a *p*-value of < 0.10 in univariate analyses were examined in the multinomial logistic regression analysis with the enter method, adjusting for age, sex, diabetes, and hypertension (Table 4). In the multivariate analysis, the use of offending drugs (odds ratio (OR) with 95% confidence interval (CI), AKD stage 3 vs. non-AKD, 3.132 (1.304–7.526), *p* = 0.011, AKD stage 2 vs. non-AKD, 2.314 (1.049–5.107), *p* = 0.038) was the independent predictor positively correlated with AKD severity. Furthermore, AKI KDIGO stage 3 (OR (95% CI), AKD stage 3 vs. non-AKD, 6.214 (2.658–14.526), *p* < 0.001) and RRT requirement within the 1st week of AKI (OR (95% CI), AKD stage 3 vs. non-AKD, 3.366 (1.008–11.242), *p* = 0.049) were also positively associated with AKD severity after adjusting for covariates (Figure 2). In summary, our analyses demonstrated that the use of offending drugs, AKI severity, and RRT requirement within the 1st week of AKI were the independent predictors of AKD severity in patients with AKI.

## 4. Discussion

In this study, we identified the use of offending drugs, AKI severity, and RRT requirement within the 1st week of AKI as the independent predictors of AKD severity. Moreover, AKD severity was positively correlated with 30-day mortality and adverse renal outcomes after AKI. In this AKI cohort composed of hospitalized patients in general medical wards, >70% of the study population progressed to AKD. The overall 30-day mortality rate was 22.90%, which increased steadily with AKD severity, and RRT-independent survival declined in a stepwise fashion with AKD severity. These findings are compatible with those of previous reports and highlight the clinical impact of AKD [11,14]. In a Chinese study, AKD with a severity of ADQI stage ≥ 2 was positively associated with 30-day mortality and RRT requirement after AKI, with 2.52 and 18.86 times the risks compared with those in non-AKD patients, respectively [12]. In another Chinese report, AKD increased approximately twice the risk of 90-day mortality after AKI [13]. A retrospective study in Lisbon also indicated that AKD aggravated short-term and long-term mortality in an AKI population with sepsis [11]. Furthermore, AKD was demonstrated to be an important risk factor for 90-day mortality in AKI patients undergoing cardiac surgery [18] and with myocardial infarction [19]. These reports and our investigation underscore the importance of AKD and the necessity to clarify its risk factors, especially those that are modifiable in clinical settings. However, the relationship between AKD severity and clinical outcomes has not been comprehensively assessed according to the standardized ADQI staging, and our study provides an instructive insight to elucidate this issue.

Although the risk factors for progression from AKI to AKD, such as age, male sex, AKI severity, etiologies of AKI, diabetes, anemia, and pre-existing kidney disease have been proposed, the predictors of AKD severity have been less studied. Hence, a comprehensive study using the standardized ADQI staging of AKD is essential [13,14]. In our analysis, the use of offending drugs was identified as an independent predictor of AKD severity, which has less been discussed in the literature. The current clinical guidelines generally recommend avoidance or dose modification of offending drugs in AKI patients. However, as a result of the population aging and increasing comorbidities, polypharmacy is extraordinarily common in real-world practice, with a prevalence of 38–50% [20,21,22]. Patients often receive multiple medications at AKI diagnosis, making an ideal drug adjustment more difficult, especially in the elderly population with multiple comorbidities. Furthermore, the spectrum of possibly relevant agents is extremely wide and heterogenous, including analgesic and anti-inflammatory agents, antihypertensives, antineoplastic agents, and nephrotoxic antibiotics [8,23,24]. For instance, NSAIDs are widely prescribed for pain relief and anti-inflammation and could exacerbate renal insufficiency especially in hemodynamically unstable patients with CKD and AKI. The NSAIDs may induce glomerular hypoperfusion, renal tubular injury, interstitial nephritis, and glomerular proteinuria, thereby leading to diverse patterns of nephrotoxicity [23]. Large-scale investigations have demonstrated that the risk of AKI in adult patients taking NSAIDs was dose-dependent, and the estimated risk of AKI with NSAID exposure has increased approximately two-fold in the literature, which could be even higher in the CKD and elderly populations [25,26]. Despite cyclooxygenase-2 selectivity and it being considered safer than traditional NSAIDs, associations between AKI and COX-2Is have been reported. Consequently, avoidance or dose adjustment of COX-2Is is generally suggested in the setting of AKI [27,28]. Because of their extensive use and hemodynamic effects on efferent arteriolar vasodilation, ACEIs and ARBs are also agents of concern in AKI. Careful evaluation or even temporary suspension of these agents is warranted during AKI period, particularly in hemodynamically unstable patients [29,30]. In a randomized trial of CKD patients undergoing cardiac catheterization, holding ACEI or ARB was associated with non-significant reduction in AKI risk (10.9% vs. 18.4%, *p* = 0.16) [31]. On the other hand, a recent prospective cohort study suggested that the continuous use of ACEI or ARB during AKI was not associated with AKD, though the study did not adhere to the ADQI definition of AKD [32]. In addition, the current evidence suggests that resuming ACEI or ARB after AKI recovery may reduce mortality risk, recurrent AKI, and incident CKD [33]. In view of the theoretical risk of eGFR decline in AKI as well as the long-term benefits to cardiovascular and renal diseases, the use of ACEI or ARB during AKI period should be adjusted individually. Furthermore, some antimicrobial agents, such as aminoglycosides and colistin, and several antineoplastic agents, such as cisplatin, methotrexate, and cancer immunotherapy, have established nephrotoxicity or possible renal complications [24,34]. Cautious evaluation of dosage or regimens of these drugs is crucial during AKI. Despite the abundant literature on AKI, there has been limited evidence specifying the role of offending drugs in AKD severity. In an analysis of patients with sepsis, He et al. (2021) suggested the use of nephrotoxic agent as a predictor of AKD initiation, but the effect on the AKD severity was not assessed [35]. In our study, the use of offending drugs was associated with over three times the risk of AKD stage 3, and over two times the risk of AKD stage 2, which highlights the impact of this modifiable risk factor and the importance of medication adjustment and monitoring during AKI. The findings will be crucial in the management of this vulnerable population, especially in those with polypharmacy and multimorbidity.

In our analyses, AKI severity was identified as an independent predictor of AKD severity, and AKI KDIGO stage 3 increased the risk of AKD stage 3 up to six times. In line with our findings, the current literature supports the associations between AKI severity and adverse outcomes such as CKD progression and long-term mortality [36,37,38], and similar correlation has been recognized in AKD recently. In a Chinese study, higher AKI KDIGO stage was a major risk factor for AKD [12], and the association was also reported in AKI patients with sepsis [35]. Previous studies identified that oliguria and peak creatinine level, which also reflect the severity of AKI, were the risk factors for AKD [14,19]. Altogether, these investigations and our research underscore the correlations between initial AKI severity and the risk and severity of AKD and, therefore, the necessity for timely management of AKI.

Currently, there is limited information about the correlation between early RRT requirement and following AKD severity. Although the initiation of RRT usually implies severe AKI, RRT may also be initiated for other indications, such as severe hyperkalemia, fluid overload, metabolic acidosis, poisoning, and renal support in critically ill patients, regardless of AKI severity [8]. Despite its efficacy in the treatment of AKI-related complications, previous reports indicated that the effect of RRT on renal recovery after AKI might be controversial [36,39,40]. In a study of critically ill patients with AKI, mortality was significantly higher in patients receiving RRT after adjusting for disease severity [41]. In addition, higher RRT intensity, including intermittent dialysis with higher frequency and continuous RRT with higher dosage, has been reported to delay renal recovery after AKI [42]. In our analysis, the RRT requirement within the 1st week of AKI elevated the risk of AKD stage 3 up to three-fold. To the best of our knowledge, there are no previous studies describing the association between early RRT requirement and AKD severity, and our findings suggest that the initiation of RRT in AKI patients should be individualized.

Our study has some limitations. Although the incidences of AKD and 30-day mortality in our investigation were compatible with those in previous studies (27–70% and 10–25% respectively) [11,12,13,14,35,43], the study cohort was retrospectively recruited from the database of the AKI e-Alert system, and AKI diagnosis was largely dependent on clinical practice. As a result, patients with AKI KDIGO stage 1 accounted for a small portion of the cohort, which was also noted in similar studies and probably related to the less frequent blood sampling in those with milder AKI [14]. Similarly, the identification of subacute AKD (i.e., AKD stage 0 of the ADQI criteria) [10] requires additional evidence of subtle renal injury, such as persistent proteinuria and other abnormal renal biomarkers, and hence could not be adequately assessed in this retrospective study. In addition, the predictive roles of oliguria, anuria, and body weight change on AKD severity were not individually assessed in our research because urine output and body weight profiles were highly unreliable in a retrospective study of patients treated in general wards. Further large-scale prospective studies utilizing other novel renal biomarkers, such as neutrophil gelatinase-associated lipocalin and kidney injury molecule-1 [44,45,46], are required to evaluate these specific issues. Although the use of antineoplastic agents and nephrotoxic antibiotics increased with AKD severity in the analysis, the impact of specific classes of offending drugs on AKD severity was not suitable to be evaluated with our current study design, which was limited by the sample size. In addition, age, sex, and pre-existing diseases or conditions, such as CKD, diabetes, main AKI etiologies, and heart failure, were not independently correlated with AKD severity in our analysis, possibly due to the overwhelming influence of AKI severity and the high average age of the cohort. Finally, all enrolled patients were of Asian origins because of the single-center nature of the study. Despite these limitations and further verifications warranted, our analysis highlights the predictive roles of offending drug usage, initial AKI severity, and early RRT requirement on AKD severity stratified by standardized ADQI staging and, therefore, will be a valuable and informative reference for clinicians and investigators to elucidate the nature and treatment strategy of AKD.

## 5. Conclusions

Our study demonstrated that the use of offending drugs, AKI severity, and RRT requirements within the 1st week of AKI were the independent predictors of AKD severity. In addition, AKD severity was positively associated with 30-day mortality and adverse renal outcomes after AKI. More research is recommended to further enhance our understanding and management of AKI and AKD.

## Figures and Tables

**Table 1:** Characteristics of patients with AKI (*n* = 310).

Demographic Profiles and Comorbidities	
Age (years), median (IQR)	69 (58–79)
Female, *n* (%)	125 (40.32)
BMI (kg/m^2^), median (IQR)	24.73 (21.30–28.73)
Baseline SCr (μmol/L), median (IQR)	83.10 (60.11–127.30)
Baseline eGFR (mL/min/1.73 m^2^), median (IQR)	71.65 (41.76–105.43)
Diabetes, *n* (%)	135 (43.55)
Hypertension, *n* (%)	163 (52.58)
Dyslipidemia, *n* (%)	47 (15.16)
CKD, *n* (%)	129 (41.61)
Heart Failure, *n* (%)	54 (17.42)
Vascular Disease, *n* (%)	78 (25.16)
Liver Disease, *n* (%)	93 (30.00)
Pulmonary Disease, *n* (%)	36 (11.61)
Malignancy, *n* (%)	142 (45.81)
Main Causes of AKI	
Sepsis, *n* (%)	188 (60.65)
Cardiorenal Syndrome, *n* (%)	22 (7.10)
Hypovolemia, *n* (%)	13 (4.19)
Obstructive Uropathy, *n* (%)	13 (4.19)
Intrarenal Causes, *n* (%)	74 (23.87)
Hematologic and Biochemical Profiles at Diagnosis of AKI	
SCr (µmol/L) at AKI Diagnosis, median (IQR)	228.96 (151.16–375.70)
eGFR (mL/min/1.73 m^2^) at AKI Diagnosis, median (IQR)	22.53 (12.06–35.38)
Hemoglobin (g/L), median (IQR)	100.00 (88.00–117.00)
Platelet (10^9^/L), median (IQR)	169.50 (101.25–253.75)
BUN (mmol/L), median (IQR)	19.99 (12.50–29.27)
K (mmol/L), median (IQR)	4.20 (3.60–4.90)
Albumin (g/L), median (IQR)	29.00 (25.00–33.90)
Lactate (mmol/L), median (IQR)	1.84 (1.29–3.46)
pH, median (IQR)	7.39 (7.30–7.45)
HCO_3_ (mmol/L), median (IQR)	21.05 (17.10–25.38)
Bil-T (µmol/L), median (IQR)	17.10 (10.26–51.31)
RRT Status and 30-Day Mortality after AKI	
RRT within 0–6 Days After AKI, *n* (%)	40 (12.90)
RRT within 7–14 Days After AKI, *n* (%)	38 (12.26)
30-Day Mortality After AKI, *n* (%)	71 (22.90)

AKI, acute kidney injury; BMI, body mass index; Bil-T, total bilirubin; BUN, blood urea nitrogen; CKD, chronic kidney disease; eGFR, estimated glomerular filtration rate; HCO_3_, blood bicarbonate; IQR, interquartile range; K, serum potassium; *n*, number; RRT, renal replacement therapy; SCr, serum creatinine.

**Table 2:** Factors Associated with AKD Severity in patients with AKI.

	Non-AKD (*n* = 71)	AKD Stage 1 (*n* = 73)	AKD Stage 2 (*n* = 95)	AKD Stage 3 (*n* = 71)	*p*-Value
Demographic Profiles and Comorbidities, *n* (%) or Median (IQR)					
Age (years)	68 (60–78)	66 (56–77)	71 (60–80)	68 (56–81)	0.437
Female	27 (38.03)	30 (41.10)	41 (43.16)	27 (38.03)	0.886
BMI (kg/m^2^)	24.62 (20.35–27.89)	25.06 (20.97–29.52)	24.98 (22.42–29.18)	24.27 (22.08–28.53)	0.408
Diabetes	26 (36.62)	37 (50.68)	46 (48.42)	26 (36.62)	0.158
Hypertension	34 (47.89)	40 (54.79)	54 (56.84)	35 (49.30)	0.621
Dyslipidemia	11 (15.49)	11 (15.07)	15 (15.79)	10 (14.08)	0.992
CKD	25 (35.21)	40 (54.79)	41 (43.16)	23 (32.39) ^b^	0.029 *
Heart Failure	11 (15.49)	20 (27.40)	18 (18.95)	5 (7.04) ^b^	0.013 *
Vascular Disease	16 (22.54)	23 (31.51)	24 (25.26)	15 (21.13)	0.487
Liver Disease	18 (25.35)	21 (28.77)	30 (31.58)	24 (33.80)	0.709
Pulmonary Disease	9 (12.68)	7 (9.59)	9 (9.47)	11 (15.49)	0.607
Malignancy	36 (50.70)	28 (38.36)	43 (45.26)	35 (49.30)	0.444
Main Causes of AKI, *n* (%)					
Sepsis	39 (54.93)	45 (61.64)	60 (63.16)	44 (61.97)	0.728
Cardiorenal Syndrome	5 (7.04)	8 (10.96)	6 (6.32)	3 (4.23)	0.453
Hypovolemia	7 (9.86)	1 (1.37)	3 (3.16)	2 (2.82)	0.052 ^#^
Obstructive Uropathy	2 (2.82)	2 (2.74)	4 (4.21)	5 (7.04)	0.541
Intrarenal Causes ^††^	18 (25.35)	17 (23.29)	22 (23.16)	17 (23.94)	0.989
Aggravating Factors at Diagnosis of AKI, *n* (%)					
Shock Status	12 (16.90)	10 (13.70)	7 (7.37)	10 (14.08)	0.286
Offending Drugs ^†^	12 (16.90)	24 (32.88)	33 (34.74)	26 (36.62) ^a^	0.038 *
Contrast Exposure	9 (12.68)	15 (20.55)	22 (23.16)	13 (18.31)	0.385
AKI Severity and RRT Status at Diagnosis of AKI, *n* (%)					
AKI KDIGO Stage 1–2	60 (84.51)	69 (94.52)	69 (72.63) ^b^	30 (42.25) ^a,b,c^	<0.001 *
AKI KDIGO Stage 3	11 (15.49)	4 (5.48)	26 (27.37) ^b^	41 (57.75) ^a,b,c^	<0.001 *
RRT within 0–6 Days	6 (8.45)	11 (15.07)	8 (8.42)	15 (21.13)	0.057 ^#^

Comparisons were performed using the chi-square test for categorical variables or the Kruskal–Wallis H-test for continuous variables. ACEI, angiotensin-converting enzyme inhibitor; AKD, acute kidney disease; ARB, angiotensin receptor blocker; COX-2I, cyclooxygenase-2 inhibitor; KDIGO, Kidney Disease Improving Global Outcomes criteria; NSAID, non-steroidal anti-inflammatory drug. ^†^: including NSAIDs or COX-2Is (*n* = 29), antineoplastic agents or nephrotoxic antibiotics (*n* = 24; cisplatin, *n* = 7, immune checkpoint inhibitor, *n* = 2, methotrexate, *n* = 4, colistin, *n* = 4, aminoglycoside, *n* = 3, vancomycin, *n* = 2, and amphotericin B, *n* = 2), ACEIs or ARBs (*n* = 39), and other drugs with nephrotoxicity (*n* = 3); ^††^: including 3 biopsy-proven glomerulonephritis (minimal change disease, *n* = 1, focal mesangial proliferative glomerulonephritis, *n* = 1, and class IV lupus nephritis, *n* = 1), 2 clinically diagnosed rapidly progressive glomerulonephritis, and 1 nephrotic syndrome with clinical exacerbation; corticosteroid (*n* = 6), cyclophosphamide (*n* = 1), and mycophenolate mofetil (*n* = 2) were prescribed as the regimens for these patients; ^a^: significantly different compared with non-AKD; ^b^: significantly different compared with AKD stage 1; ^c^: significantly different compared with AKD stage 2; *: *p* < 0.05; ^#^: *p* < 0.10.

**Table 3:** Hematologic and biochemical profiles associated with AKD Severity in patients with AKI.

	Non-AKD (*n* = 71)	AKD Stage 1 (*n* = 73)	AKD Stage 2 (*n* = 95)	AKD Stage 3 (*n* = 71)	*p*-Value
Hematologic and Biochemical Profiles at Diagnosis of AKI, Median (IQR)					
eGFR (mL/min/1.73 m^2^)					
*Baseline*	81.31 (50.28–107.18)	56.90 (27.82–90.71)	70.18 (38.76–101.99)	85.14 (57.32–127.95) ^b^	0.003 *
*At AKI Diagnosis*	26.92 (16.00–40.00)	20.21 (12.00–34.24)	21.00 (13.00–36.70)	19.83 (9.19–32.25)	0.134
Hemoglobin (g/L)	103.00 (86.80–124.50)	98.00 (86.00–111.50)	100.50 (88.00–118.30)	99.00 (89.00–113.00)	0.380
Platelet (10^9^/L)	159.50 (87.50–259.00)	165.00 (104.00–273.50)	169.50 (107.50–255.50)	183.00 (106.00–236.00)	0.924
BUN (mmol/L)	17.49 (10.17–26.15)	21.78 (13.74–31.42)	19.99 (12.05–29.01)	19.99 (13.21–30.70)	0.592
K (mmol/L)	4.20 (3.78–4.80)	4.10 (3.60–4.65)	4.15 (3.60–5.33)	4.10 (3.70–5.00)	0.507
Albumin (g/L)	30.00 (26.90–35.00)	29.30 (24.90–33.00)	29.00 (25.40–33.00)	28.00 (24.40–33.00)	0.285
Lactate (mmol/L)	2.46 (1.37–5.13)	1.83 (1.35–2.73)	1.69 (1.08–4.75)	1.68 (1.25–2.96)	0.327
pH	7.42 (7.31–7.49)	7.41 (7.34–7.45)	7.37 (7.29–7.45)	7.37 (7.30–7.43)	0.106
HCO_3_ (mmol/L)	21.10 (17.90–25.48)	21.20 (17.30–24.10)	20.90 (16.40–27.23)	19.80 (15.80–25.30)	0.907
Bil-T (µmol/L)	17.10 (10.26–35.92)	15.39 (9.07–37.63)	18.81 (10.26–119.73)	18.81 (9.41–54.73)	0.688

All comparisons were performed using the Kruskal–Wallis H-test. ^b^: significantly different compared with AKD stage 1; *: *p* < 0.05.

**Table 4:** Independent predictors of AKD severity in patients with AKI.

	AKD Stage 1 vs. Non-AKD		AKD Stage 2 vs. Non-AKD		AKD Stage 3 vs. Non-AKD	
	Adjusted OR (95% CI)	*p*-Value	Adjusted OR (95% CI)	*p*-Value	Adjusted OR (95% CI)	*p*-Value
Age (years)	0.981 (0.957–1.006)	0.139	1.004 (0.980–1.029)	0.741	1.003 (0.976–1.031)	0.815
Female	1.155 (0.568–2.347)	0.691	1.217 (0.630–2.354)	0.559	1.141 (0.534–2.438)	0.733
Diabetes	1.347 (0.603–3.008)	0.467	1.375 (0.652–2.902)	0.403	0.928 (0.391–2.203)	0.865
Hypertension	0.796 (0.358–1.767)	0.575	0.995 (0.475–2.087)	0.990	1.094 (0.468–2.559)	0.835
CKD	1.468 (0.518–4.158)	0.470	1.449 (0.586–3.586)	0.422	1.157 (0.417–3.212)	0.779
AKI KDIGO Stage 1–2	Reference		Reference		Reference	
AKI KDIGO Stage 3	0.368 (0.108–1.263)	0.112	2.243 (0.983–5.117)	0.055	6.214 (2.658–14.526)	<0.001 *
RRT within 0–6 Days	1.480 (0.454–4.829)	0.515	0.915 (0.276–3.037)	0.885	3.366 (1.008–11.242)	0.049 *
Hypovolemia	0.130 (0.015–1.127)	0.064	0.330 (0.077–1.411)	0.135	0.265 (0.044–1.601)	0.148
Heart Failure	1.477 (0.601–3.632)	0.396	1.065 (0.438–2.589)	0.889	0.557 (0.167–1.856)	0.341
Offending Drugs	1.934 (0.827–4.522)	0.128	2.314 (1.049–5.107)	0.038 *	3.132 (1.304–7.526)	0.011 *
Baseline eGFR (mL/min/1.73 m^2^)	0.996 (0.986–1.007)	0.503	1.002 (0.994–1.010)	0.672	1.004 (0.996–1.012)	0.324

Age, sex, diabetes, hypertension, and covariates with a *p*-value of < 0.10 in univariate analyses were adjusted for multinomial logistic regression analysis with the enter method. CI, confidence interval; OR, odds ratio. *: *p* < 0.05.

## Data Availability

All data generated in this study are available from the corresponding authors (b9302095@cgmh.org.tw (K.-F.T.); tytc107@gmail.com (T.T.-Y.C.)) upon reasonable request due to research regulations of the hospital.
